# Review: Suicide and its relationship to aggression and impulsivity

**DOI:** 10.3758/s13415-025-01321-0

**Published:** 2025-06-25

**Authors:** Yoojin Lee, Jessica R. Gilbert, Laura R. Waldman, Carlos A. Zarate, Elizabeth D. Ballard

**Affiliations:** Experimental Therapeutics and Pathophysiology Branch, Intramural Research Program, National Institute of Mental Health, National Institutes of Health, Building 10, CRC Room 7-3332, 10 Center Drive, MSC 1282, Bethesda, MD 20892 USA

**Keywords:** Suicide, Aggression, Impulsivity

## Abstract

Aggression and impulsivity are key risk factors for suicide, which remains a public health concern. The heterogeneity of suicidal behaviors, combined with the complexities of aggression and impulsivity, complicates the investigation of their relationship with suicide risk. This review sought to provide a comprehensive overview of the literature examining the relationship between aggression, impulsivity, and suicide. Broadly, individuals with higher levels of aggression and impulsivity were found to be more vulnerable to suicidal behaviors. Stress, the immune system, and neurotransmitters also appeared to affect the relationship between aggression, impulsivity, and suicide. The reactive aggression and proactive aggression subtypes were each found to uniquely contribute to suicide risk. Furthermore, although different facets of impulsivity have varied relationships with suicide risk, self-reported impulsivity did not consistently align with task-driven impulsivity, and distinct facets of task-driven impulsivity demonstrated unique associations with suicide risk. Task-driven impulsivity and reward-based learning, as estimated by reinforcement learning hyperparameters, may provide valuable insights into the potential utility of tasks that assess risk factors in suicide research and their relationship with sensory and emotion regulation in the brain. In addition, neuroimaging studies indicated that decreased cognitive capability and control may be involved in the link between impulsivity and suicide. Collectively, the evidence presented herein highlights the complex interplay between aggression, impulsivity, and suicide. Understanding the underlying genetic, epigenetic, stress, neural, and neurotransmitter factors involved is crucial for developing effective prevention and intervention strategies for individuals at risk of suicide.

## Introduction

Suicide is a complex and serious public health issue. Each year, more than 700,000 people worldwide lose their lives to suicide (World Health Organization, 2021). In the United States, it ranks as the 12th leading cause of death overall and the second and third leading causes of death among adolescents and young adults, respectively (Garnett & Curtin, 2023). Furthermore, suicide rates have increased by 30% over the past 2 decades, and the impact of this increase extends well beyond the individuals who attempt or die by suicide; family members of those who die by suicide also experience lower mental and economic well-being, resulting in a net adverse effect on society (Bolton et al., 2013; Sveen & Walby, 2008).

Despite its high prevalence and profound consequences on public health, our understanding of suicidal acts and risks remains lacking. Previous research focused on identifying psychosocial factors as well as objective neurobiomarkers of suicide risk (for a review, see Carballo et al., 2020; Lamontagne et al., 2023). In this context, impulsivity and aggression have emerged as key risk factors positively correlated with risk of suicide (Zouk et al., 2006) and suicidal behaviors (Doihara et al., 2012; Gvion et al., 2014; Hadzic et al., 2020; Mann, 2003; Swann et al., 2005). Aggression is characterized by the manifestation of harmful behaviors toward others, while impulsivity is defined by the tendency to act on urges without considering the consequences. Individuals with higher levels of aggression and impulsivity have been found to be more vulnerable to self-harm, suicidal thoughts, suicide attempts, and completed suicide (Auerbach et al., 2017; Ben-Efraim et al., 2011; Cha et al., 2018; McGirr et al., 2008; Orri et al., 2020). However, it is important to note that while aggression and impulsivity are interrelated constructs, they are distinct in nature. Although these traits are highly correlated in general, their subfactors reveal variations in the relationship between aggression and impulsivity (García-Forero et al., 2009). For example, impulsivity is strongly associated with reactive aggression but not with proactive aggression (Ramirez & Andreu, 2006). The lack of conceptual clarity and cohesion among measures of aggression and impulsivity remains limited, which complicates effort to explore their relationship with suicide risk. Moreover, sex-specific mechanisms may introduce additional complexities when investigating the association between impulsivity, aggression, and suicidal behavior.

This review sought to delineate the various subtypes of these related but distinct constructs and clarify their association with suicide risk. Understanding the association between these factors is pivotal for developing effective prevention strategies and interventions for at-risk individuals. The review will examine key aspects related to suicide, including

1. The stress-diathesis model of aggression and impulsivity in the context of suicide;
2. The significance of aggression and impulsivity and their subtypes as risk factors for suicide;
3. The potential utility of reflection impulsivity and value-based learning on suicide in addition to their temporal dynamics;
4. Genetic, epigenetic, and stress markers for aggression and impulsivity in the context of suicide; and
5. Potential avenues for enhancing suicide research.

The review seeks to expand our understanding of aggression and impulsivity as general constructs as well as suicide risk subtypes in ways that will help the field assess the current state of research, identify areas for improvement, and ultimately help identify at-risk individuals.

### The stress-diathesis model for aggression and impulsivity

Mann’s stress-diathesis model is a neurocognitive model that seeks to explain the complex interplay between predisposing cognitive behavioral factors (diatheses) and external stressors that contribute to suicidal behavior (Mann, 2003). Under this model, genetic predisposition and epigenetic modification induced by life stressors through DNA methylation contribute to dysregulation of the hypothalamic-pituitary-adrenal (HPA) axis and the immune system. This dysregulation manifests as neurotropic/apoptotic deficits and neuroinflammation. The dysregulated stress and immune systems may, in turn, dysregulate emotional response and disrupt neural reactivity and connectivity in brain regions, including the frontal cortices, inferior frontal gyrus, and insula. Such systems also affect neurotransmitter function, including serotonin, catecholamines, glutamate, and gamma aminobutyric acid (GABA). These disrupted neural and neurotransmitter activities subsequently prompt diatheses such as subjective distress, impaired executive functioning (e.g., decision-making), learning/memory deficits, social distortion, and sleep deprivation (O’Connor et al., 2020). Diatheses interact with both internal (e.g., mental illness-related) and external (e.g., financial, social) stressors, and the interaction ultimately manifests as suicidal behavior.

Collectively, the stress-diathesis model proposes that genetic predisposition and epigenetic modifications caused by life stressors contribute to dysregulation of the stress response system (HPA axis and immune system) and to neurotransmitter function (serotonin, catecholamines, glutamate, GABA), leading to the diatheses that interact with stressors and increase suicide risk. Importantly, aggression and impulsivity are known risk factors for suicide (Gvion & Apter, 2011; Stanley et al., 2019a), and these traits are hypothesized to influence suicide risk by disrupting key neural circuits and neurotransmitter systems (Mann & Rizk, 2020). However, it should be noted that the exact mechanisms through which aggression and impulsivity contribute to suicidal behaviors remain unclear, and it is uncertain whether they are prerequisites for suicidal behaviors. It is also important to note that the stress-diathesis model may fall short of capturing the dynamic, bidirectional interplay among the multiple components that influence the relationship between aggression, impulsivity, and suicide risk. This review seeks to address this challenge by offering insights into these complexities.

### Aggression and impulsivity

#### Aggression

Broadly, aggression—in combination with emotion regulation—is tied to suicide risk. Aggression is a behavioral trait characterized by actions intended to cause harm or assert dominance. It can manifest rapidly, without forethought, or through planned, deliberate action. Interestingly, recent suicide attempters reported high degrees of aggression, as measured by the Buss-Perry Aggression Questionnaire (Doihara et al., 2008). Among combatants, predeployment aggression and emotion regulation scores were found to play a moderating role in suicide risk following exposure to morally challenging events postdeployment (Levi-Belz et al., 2023). Another study found that level of aggression, as assessed by the Brown-Goodwin Lifetime History of Aggression scale, distinguished individuals with a history of suicide attempts and borderline personality disorder (BPD) from those without such a history, and that individuals who had attempted suicide displayed higher levels of aggression (Keilp et al., 2006).

Aggression has also been linked to both brain volume and activity in individuals at risk for suicide. For instance, participants with bipolar disorder and elevated aggression levels were found to have reduced gray matter volume in the orbitofrontal cortex and insula (Drachman et al., 2022), which may impair sensory processing efficiency. Another study found that resting-state magnetoencephalography (MEG) power in inpatients with recent suicide attempts or ongoing suicidal thoughts and high levels of aggression was inversely related to activity in the middle frontal gyrus, angular gyrus, inferior frontal gyrus, and precuneus (Lee et al., 2024). Such brain regions have been implicated in sensory and emotional processes as well as motivation (Gogolla, 2017; Rolls, 2023), suggesting that the neurobiological underpinnings of aggression in relation to suicide risk involve sensory and emotion-related mechanisms.

Although aggression is thought to contribute to suicide risk at the behavioral and neural level, conceptual inconsistencies across different subtypes of aggression complicate this paradigm. Aggression is often categorized based on the social context relevant to the aggressive behavior (e.g., proactive vs. reactive aggression) or individual tendencies that affect emotion regulation, especially in humans (e.g., premeditated vs. impulsive aggression; Smeijers et al., 2018; Volicer, 2021; Wrangham, 2018). Proactive aggression refers to the strategic use of aggressive behaviors to achieve personal benefits, such as intimidating individuals of lower status, goal-directed behaviors, callous-unemotional traits, or antisocial behaviors (Frick & White, 2008; Vaughan et al., 2024). In contrast, reactive aggression is a nonpremeditated response triggered by pressure or provocation, such as attacking in response to a perceived threat, hostility, motor impulsivity, or negative affect (Bertsch et al., 2020; Fite et al., 2009). Proactive aggression is often used interchangeably with premeditated aggression, whereas reactive aggression is equated with impulsive aggression.

It is worth noting that considerable overlap exists between these subtypes, complicating distinctions (Babcock et al., 2014). For example, adolescents with *both* higher reactive and proactive aggression had lower empathy than those with *either* high reactive or proactive aggression, suggesting a synergistic relationship between the two (Euler et al., 2017). The overlap may stem from shared genetic and environmental influences. Genetic studies indicate that reactive and proactive aggression share substantial genetic contributions (26–45%), with minimal subtype-specific genetic variance (<11%; Brendgen et al., 2006; Romero-Martinez et al., 2022). Nonshared environmental influences also contribute to their intercorrelation (Card & Little, 2006; Tuvblad et al., 2009). In addition to genetic and environmental influences, previous studies found that both reactive and proactive aggression were positively correlated with medial prefrontal cortex (mPFC) activity, while lateral prefrontal cortex stimulation inversely correlated with aggression, regardless of subtype (Card & Little, 2006; Romero-Martinez et al., 2022; Smith et al., 2018; Tuvblad et al., 2009). These neural findings highlight the role of brain regions involved in attention and emotion regulation (lateral and medial PFC) in the relationship between aggression and suicide risk and underscore the importance of developing refined frameworks to differentiate between aggression subtypes and their distinct contributions to suicide risk.

Research has shown that reactive aggression is associated with suicide risk, including a history of suicide attempts as well as suicidal thoughts and behaviors (Conner et al., 2009; Hartley et al., 2018; Ricarte et al., 2022; Swogger et al., 2014b); however, its relationship to another suicidal marker, impulsivity, is nuanced (Abel et al., 2020; Cervantes & Delville, 2007). For example, individuals with both BPD and major depressive disorder (MDD) exhibited more frequent lifetime suicide attempts and higher levels of aggression and hostility, primarily triggered by interpersonal conflicts, than those with MDD alone (Brodsky et al., 2006). Symptoms of increased reactive aggression behaviors, such as careless acts and anger toward threats, were significantly more pronounced in individuals with these comorbid conditions. This relationship between increased suicide risk and reactive aggression may be due to dysfunction of emotional regulation (Long et al., 2014). In addition, McCloskey and Ammerman (2018) surmised that suicide attempts and the associated risk of mortality were likely related to reactive aggression- and impulsivity-related disorders, including conduct disorder and BPD, somewhat independently from other psychopathological traits (McCloskey & Ammerman, 2018).

Reactive aggression may be linked to emotion- and sensory-regulating mechanisms. When faced with situations where escape is impossible, individuals often demonstrate aggressive approaches rather than avoidance (Carver & Harmon-Jones, 2009). This expression of anger can result in challenges related to regulating emotions and the ability to exert inhibitory control, both of which are associated with the brain’s reward mechanisms (Blair, 2016). The expression of anger is also influenced by internal states such as hunger, energy levels, reproductive state, stress, and diurnal rhythms as well as past experiences (Lischinsky & Lin, 2020). In short, reactive aggression appears to be linked to suicide risk though emotion regulation and impulsivity and to be mediated by internal energy metabolism and biological rhythms, especially in challenging environments perceived as inescapable.

Proactive aggression is characterized by planned and goal-oriented behaviors, executed for a reward and accompanied by low autonomic arousal (Conner et al., 2003). This type of aggression has been associated with suicidal ideation and attempts. A large population study revealed that reactive aggression, which is more impulsive and emotionally driven, is also linked to suicidal thoughts and attempts, with this association being particularly pronounced in males (Conner et al., 2009). However, follow-up studies suggest a more nuanced picture. For instance, reactive aggression was not associated with suicide risk among criminals (Swogger et al., 2014b). Outwardly directed aggression, which involves expressing aggression towards others and objects, has been positively associated with planned, rather than unplanned, suicide attempts (Swogger et al., 2014a). This suggests that the relationship between proactive aggression and suicide attempts may be mediated by context, such as the target of the anger.

Although both reactive and proactive aggression are linked to suicide risk, they are etiologically distinct. Reactive aggression in animals is often studied using the “resident-intruder” paradigm, while proactive aggression is associated with social hierarchy and resource availability (Kwiatkowski et al., 2021; Shemesh et al., 2024). Some researchers have suggested that reactive aggression stems from defensive mechanisms, whereas proactive aggression is predatory (Battivelli et al., 2024; Potegal & Nordman, 2023); notably, however, animals exhibit proactive aggression less frequently than reactive aggression and less frequently than humans. Distinguishing between reactive and proactive aggression in humans is challenging but important. For instance, in the go/no-go task, adolescents with high reactive aggression showed smaller no-ho P3 and ERN amplitudes following emotional facial expressions (Sun et al., 2020, 2023). Interestingly, monetary rewards in the Taylor Aggression Paradigm (TAP)—which has been used to elicit aggression—can induce reactive aggression-like responses (Konzok et al., 2020). Males tend to display more reactive aggression during the TAP but report higher proactive aggression on the Reactive-Proactive Aggression Questionnaire (RPQ; Dambacher et al., 2015; Konzok et al., 2020). Unprovoked, males show higher levels of reactive aggression than females, but this metric increases in females under provocation (Boccadoro et al., 2021). Individuals with high trait-like proactive aggression have also shown increased proactive aggression after losing during the TAP (Boccadoro et al., 2024). However, it is critical to note that self-reported measures of aggression do not always align with behavior observed in the TAP or with physiological arousal measured by skin conductance (Boccadoro et al., 2024). This relationship varies by biological sex, with females showing increased physiological arousal at higher proactive aggression levels, while males show decreased arousal (Boccadoro et al., 2021). These findings underscore the need for further empirical validation to understand the mechanisms underlying these forms of aggression and their link to suicide risk.

Given these discrepancies, it is important to distinguish aggression subtypes when examining their potential roles in suicide risk. A theory proposed by Stanley and colleagues outlines two distinct trajectories via which reactive and proactive aggression may contribute to suicide risk (Stanley et al., 2021). The first trajectory (i.e., the stress driven reactive aggression pathway) posits that reactive aggression, coupled with heightened cortisol responses to stress, impaired cortical control, and early childhood adversity, predicts fluctuations in suicidal thoughts, particularly following adverse life events. This pathway suggests that early childhood adversity impacts suicidal thoughts indirectly through its influence on reactive aggression, dysregulated cortisol responses, and poor emotional and cognitive control, with these mediating factors activated by significant life stressors. The second trajectory (i.e., low reactive aggression pathway) suggests that individuals with intact cognitive control and elevated serotonin receptor binding potential may still exhibit suicidal thoughts and behaviors, often triggered by depressive episodes. In this pathway, low reactive aggression is associated with cognitive control and serotonin receptor binding potential.

The distinction between high reactive and low reactive (or high proactive) aggression is central to understanding these pathways. Empirical evidence supports the stress-driven reactive aggression pathway, particularly the role of early childhood adversity in shaping subsequent suicide risk. Studies have linked childhood adversity to suicidal thoughts and attempts (Carbone et al., 2021; Stansfeld et al., 2017; Thompson et al., 2019). Childhood adversity has also been associated with deficits in cognitive control, as demonstrated in tasks such as the emotional Stroop, go/no-go, and Simon tasks (Lambert et al., 2017; Machlin et al., 2019; Quinn et al., 2018). Such adversity also disrupts neural circuits underlying emotional regulation and cognitive control (Duffy et al., 2018), increases reactive aggression (Perry et al., 2021), and dysregulates HPA axis reactivity during acute stress tasks like the Trier Social Stress Test (TSST; Busso et al., 2017; Counts et al., 2022; Kuras et al., 2017; Simon & Admon, 2023). These findings suggest that impaired emotional and cognitive control, heightened reactive aggression, and dysregulated cortisol responses resulting from childhood adversity may collectively contribute to suicidal ideation and behaviors (Brokke et al., 2020; Conner et al., 2009; Hartley et al., 2018; Hatkevich et al., 2019; Lee et al., 2023; Melhem et al., 2016; O’Connor et al., 2017; Stanley et al., 2019a). The stress-driven reactive aggression pathway thus presents a promising framework for future research.

In contrast, evidence for the low reactive aggression pathway remains less supported. While serotonin receptor binding in the ACC and dorsal raphe nucleus is known to moderate emotional and fear responses (Bocchio et al., 2016; da Cunha-Bang et al., 2017; Janet et al., 2023; Marcinkiewcz et al., 2016), its role in enhancing cognitive control or reducing reactive aggression is less clear. Evidence linking proactive aggression or reactive aggression to suicide risk appears to be subtype specific and may involve nonlinear relationships; these varied findings highlight the need for empirical validation of both the proactive and reactive aggression pathways and their proposed mechanisms. Thus, while the model proposed by Stanley and colleagues (2021) provides a foundation, more research is needed to unravel the intricate interactions between proactive aggression, cognitive regulation, serotonergic function, and suicidal behavior, particularly from a neuropsychological perspective.

#### Impulsivity

Impulsivity, characterized by a tendency to act on urges without fully comprehending the consequences, is recognized as a context-dependent multifaced construct, which complicates its relationship with clinical phenotypes (Strickland & Johnson, 2021). Ongoing debates and disagreements persist regarding the relationship between impulsivity and suicidal behavior (Minzenberg et al., 2015) despite extensive research exploring the association between the two (Doihara et al., 2012; Ghanem et al., 2013). The varied contextual meanings assigned to impulsivity in previous studies contribute to these ongoing disagreements. For instance, one meta-analysis found that specific subtypes of impulsivity, such as response inhibition and risky decision-making, showed stronger associations with self-harm and suicidality, whereas others, like delay discounting and cognitive inhibition, did not (McHugh et al., 2019). Interestingly, the subtypes did not moderate the relationship between impulsivity and self-harm or suicidal behaviors. These findings underscore how heterogeneity in the definitions of impulsivity and suicidal behavior complicate the relationship between impulsivity and suicide risk.

Impulsivity can be measured through self-reported questionnaires as well as behavioral tasks. Previous research defined impulsivity by using questionnaires that measure an individual’s self-perceived tendency to act impulsively, including Eysenck’s Personality Questionnaire (Eysenck & Eysenck, 1975), the Dickman Impulsivity Instrument (Dickman, 1990), the Behavioral Inhibition/Activation System (BIS/BAS; Carver & White, 1994), the Urgency, Premeditation, Perseverance, Sensation Seeking (UPPS) Impulsive Behavior Scale (Whiteside & Lynam, 2001), and the Barratt Impulsivity Scale (BIS; Barratt, 1959; Stanford et al., 2009). It should be noted that impulsivity, as assessed by these questionnaires, is typically treated as a trait-like construct. However, this construct is heterogeneous and encompasses specific subtypes, possibly capturing different aspects of impulsivity; thus, researchers should be cautious about both the contextual specificity of impulsivity and its interpretation as measured via different questionnaires.

Most studies that used self-reported impulsivity metrics, which are aggregated over a long period of introspection, found a positive relationship between impulsivity and suicide risk, including suicidal thoughts and a history of suicide attempts (Doihara et al., 2012; Klonsky & May, 2010; Kumar et al., 2013; Lynam et al., 2011; Ponsoni et al., 2018; Teh et al., 2023). These relationships can be complex and depend on the specific impulsivity subtype (Gao et al., 2011). As a salient example, research with the BIS/BAS suggested that the link between impulsivity, suicidal thoughts, and a history of suicide attempts was mediated via self-reflection (Khosravani et al., 2020). Elevated BIS scores are associated with higher suicide risk, including a history of suicide attempts and suicidal thoughts, in adults with MDD (Ekinci et al., 2011; Öğüt et al., 2023). These elevated scores could potentially lead to future suicidal acts (Oquendo et al., 2004). In addition, high scores on the urgency and/or lack of premeditation subscales of the UPPS—which indicate heightened emotional impulsivity and impaired premeditative thinking—are also linked to suicide risk, particularly suicidal thoughts (Bruno et al., 2023; Klonsky & May, 2010; Lynam et al., 2011; Picou et al., 2024). However, a study of college students found no evidence supporting the association between these UPPS subscales and self-injurious behavior (Glenn & Klonsky, 2010), suggesting that these subscales may more effectively distinguish suicidal thoughts rather than non-suicidal self-injury. This finding highlights the positive relationship between self-reported impulsivity and suicide risk and the importance of context when interpreting the relationship between self-reported impulsivity measures and suicide risk.

Few studies have investigated neural correlates associated with the relationship between self-reported impulsivity and suicide. One study found that impulsivity, as measured by the BIS, was associated with an increase in connectivity metrics in the posterior cingulate cortex in veterans with a history of suicide attempts (Hwang et al., 2018) and with a decrease in white matter volume in the anterior cingulate cortex (ACC; Huber et al., 2021), indicating that regions implicated in cognitive control processes might contribute to suicide risk (Leech et al., 2012; Monosov et al., 2020). A recent study from our laboratory investigated the neural correlates of impulsivity, as measured by the BIS, in inpatients with recent suicide attempts or ongoing suicidal thoughts (Lee et al., 2024); resting-state MEG power was found to be inversely related to activity in several brain regions, including the middle frontal gyrus, angular gyrus, supramarginal gyrus, inferior parietal lobule, precuneus, and postcentral gyrus. In addition, dynamic causal modeling results showed that impulsivity was negatively related to α-amino-3-hydroxy-5-methyl-4-isoxazolepropionic acid (AMPA)-driven glutamatergic connectivity from the precuneus to the insula. These results suggest that deficits in neural processes associated with cognitive control and emotion regulation may serve as a potential marker for understanding the relationship between self-reported impulsivity and suicide risk. However, given the few studies that have investigated neural correlates of impulsivity in the context of suicide, more research is needed to validate these findings.

Broadly, studies suggest that self-reported impulsivity is positively associated with suicide risk in general. For example, in individuals with depression, a history of suicide attempts has been linked to the attentional and nonplanning subtypes of the BIS (Ekinci et al., 2011). Suicidal thoughts are also associated with total BIS score and with the attentional BIS score subtype (Öğüt et al., 2023). Individuals with previous suicide attempts tend to have higher total and subscale BIS scores than healthy volunteers, although there was no significant difference in BIS scores between individuals with previous suicide attempts and those with suicidal thoughts (Hwang et al., 2018). However, recent suicidal crises are associated with increased BIS scores across all subscales compared with individuals with previous suicide attempts, mood disorders only, and healthy volunteers (Lee et al., 2024), indicating that the severity of suicide risk may affect its relationship to impulsivity, particularly when temporal dynamics are taken into account. On the UPPS, previous suicidal thoughts and attempts were found to be positively associated with urgency, whereas only previous suicide attempts were related to premeditation (Klonsky & May, 2010). Increased negative urgency appears to be a particularly promising marker for suicidal thoughts and attempts, potentially mediated by perceived burdensomeness and thwarted belongingness (Bruno et al., 2023; Lynam et al., 2011; Picou et al., 2024). Together, these findings suggest that suicide risk is positively related to total and/or subscale BIS scores and UPPS scores, particularly negative urgency, except in cases where the relationship may be nonlinear.

However, any such associations are also likely influenced by the context specific to impulsivity questionnaires—that is, what those questionnaires measure—although this limitation may be compensated for by another form of impulsivity: moment-to-moment task-based impulsivity. To unravel the complex relationship between moment-to-moment task-based impulsivity and suicide risk, this relationship can be examined through the lens of task-based impulsivity subtypes. Specifically, the schematic categorization proposed by Voon (2020) partially incorporates the frameworks recommended by Strickland and Johnson (2021). Voon’s categorization of impulsivity better captures response-based behavioral components of impulsivity but may not fully align with self-reported measures of attentional, motor, and nonplanning impulsivity from the BIS, or with negative and positive urgency, premeditation, perseverance, and sensation seeking measures from the UPPS. This discrepancy arises because self-reported scales capture past perceived behaviors or future planned behaviors in an imagined context influenced by self-perception, which may differ from moment-to-moment task-driven impulsivity. Two distinct types of moment-to-moment task-driven impulsivity have been identified: motor impulsivity and decision impulsivity (Voon, 2020). Motor impulsivity encompasses premature anticipatory responses (Tremblay et al., 2019; Voon et al., 2016) and failures in response inhibition (Bari & Robbins, 2013; Eagle et al., 2008; Pawliczek et al., 2013). In contrast, decision impulsivity involves aspects such as delay discounting, risk-taking, and reflection impulsivity. Given the uncertainty around this issue, it is crucial to establish comprehensive and precise definitions of moment-to-moment task-driven impulsivity in order to further explore their neurobiological relationship with suicidal behavior. The relationships between these distinct types of moment-to-moment task-driven impulsivity and suicide risk are explored below.

##### Motor impulsivity: Waiting impulsivity

Premature anticipatory response, also known as waiting impulsivity, refers to the inability to withhold action initiation (Chan et al., 2023). To date, no studies have directly investigated the relationship between waiting impulsivity and suicide risk. This gap in research may stem from the conceptual challenge of linking waiting impulsivity to suicide, as this form of impulsivity is closely tied to reward anticipation. The notion of suicide as a goal-oriented behavior with an anticipated reward is complex and difficult to operationalize, potentially hindering exploration of this relationship.

##### Motor impulsivity: Response inhibition

Moment-to-moment response inhibition refers to the inability to stop an ongoing response. Behavioral studies found that response inhibition during the go/no-go task was inversely correlated with suicide attempt history (Moniz et al., 2017) and with suicidal thoughts (Harfmann et al., 2019; Öğüt et al., 2023). These findings suggest that individuals at risk for suicide may exhibit impaired response inhibition and underscore the potential role of motor impulsivity in suicidal behaviors. The PFC and other frontal lobe regions have been implicated in this process (Aron, 2011). For example, adolescents with depression and a history of suicide attempts displayed reduced activation in the right anterior cingulate gyrus during a response inhibition task compared with those without a history of suicide attempts (Pan et al., 2011).

Few studies have examined emotion inhibition and suicidal markers using electrophysiological methods. One study found that individuals with a history of suicide attempts and suicidal crisis had reduced P3d amplitude, a quasi-index of emotion inhibition, in response to emotional stimuli (Porteous et al., 2023; Tavakoli et al., 2022). Response inhibition and its relationship to history of suicide attempts has also been investigated in individuals with depression. During the no-go portion of the go/no-go task, individuals with a history of suicide attempts showed a decoupling between the alpha and beta bands in brain regions including the dorsal ACC and ventral PFC (Dai et al., 2022) as well as increased precentral gyrus activity (Gifuni et al., 2024), suggesting potential malfunctions in these brain regions related to motor and cognitive control. However, one study investigating the association between response inhibition and a history of suicide attempts found that girls with depression showed no differences in response inhibition based on their history of suicide attempts or the frequency of these attempts (Mathias et al., 2011), suggesting that biological sex may be a potential covariate. These findings suggest the possible involvement of specific brain regions, such as the anterior cingulate gyrus, primary and supplementary motor cortices, and posterior cingulate cortex, in the failure of response inhibition and delayed response to cues. These neural dysfunctions in brain regions such as the ACC and PFC may be precursors to suicidal behaviors, including suicide attempts. This impairment may reflect broader deficits in cognitive control processes, highlighting the need for further research into inhibitory mechanisms as potential biomarkers of suicide risk.

##### Decision impulsivity: Delay-discounting impulsivity

Delay discounting impulsivity refers to the tendency to prefer immediate, smaller rewards over larger, delayed rewards. It has been associated with various psychopathologies, including depression (Levitt et al., 2023). The relationship between suicide attempt history and delay discounting is complex. Some studies have shown a positive relationship between delay discounting and a history of suicide attempts (Bryan & Bryan, 2021; Fang et al., 2022), nonsuicidal self-injuries (Dougherty et al., 2009), and frequency of past suicide attempts (Mathias et al., 2011). Delay discounting has also been negatively associated with brain volume, particularly in the putamen (Dombrovski et al., 2012). However, other studies found no direct association between these factors (Bridge et al., 2015; Tsypes et al., 2022), suggesting that the association may also be influenced by third factors, such as the presence of posttraumatic stress disorder (PTSD; Bryan & Bryan, 2021) or social context (Fang et al., 2022).

Neuroimaging studies found that delay discounting was associated with increased brain activity in frontal, parietal, and temporal cortical regions, as well as subcortical regions such as the thalamus and striatum (Dombrovski & Hallquist, 2017; Frost & McNaughton, 2017). This heightened activity is thought to be influenced by dopaminergic function, particularly in the PFC and ventral striatum (Joutsa et al., 2015; Pine et al., 2010). In terms of functional connectivity, delay discounting is positively related to connectivity within the default mode network and negatively related to connectivity between the dorsal PFC and the ventral attention network (Mehta et al., 2023). However, in individuals with a history of suicide attempts, deactivation of the lateral PFC was observed in response to value differences favoring the immediate option, while diminished activation in the left parahippocampal gyrus and left middle occipital gyrus was seen while favoring the delayed option (Vanyukov et al., 2016). A recent study from our laboratory found no association between suicide risk and delay discounting, as measured by the Monetary-Choice Questionnaire (MCQ; Lee et al., 2024). Thus, the relationship between delay discounting and suicidal behavior appears to be inconsistent and multifaceted. Neural correlates underlying delay discounting, such as attentional processes (involving the parahippocampal and occipital gyrus) and regulatory functions (mediated by the lateral PFC), may play a role in shaping this association. In addition, comorbid conditions like PTSD could act as modulators, further complicating any interpretation of these findings. These complexities highlight the need for further research to disentangle the neural and psychological factors influencing delay discounting in individuals at risk for suicide.

##### Decision impulsivity: Risk-taking impulsivity

Risk-taking impulsivity describes a propensity to engage in actions that involve significant risk of negative outcomes without considering potential consequences or pursuing the outcome at the expense of risk. While limitations exist, risk-taking impulsivity has been implicated in suicide risk (Ammerman et al., 2018; Dougherty et al., 2009) and as a partial mediator of the relationship between alcohol use, depression, and suicidal thoughts (Liu et al., 2022). However, the role of risk-taking impulsivity in explaining suicidal behavior is more intricate. For instance, studies using a monetary gambling task found that individuals with a history of suicide attempts showed characteristics of both high risk and loss aversion (Baek et al., 2017; Liu et al., 2022), which may protect them from potential harm. However, another study found no association between risk-taking impulsivity and suicidal thoughts (Hadlaczky et al., 2018), suggesting that the relationship is complex and may vary depending on the specific behavioral context in which the risk-taking is measured.

Moment-to-moment risk-taking propensity, measured by performance on the BART, has been linked to increased brain activity in subcortical regions such as the thalamus and dorsal striatum (Panwar et al., 2014) as well as enhanced cognitive flexibility, as indicated by lower Hurst exponential scores in the inferior frontal gyrus and anterior insula (Gentili et al., 2020). Conversely, higher BART scores have been associated with reduced spontaneous neural activation in executive function regions, as reflected by lower amplitude of low-frequency fluctuations in the ACC and mPFC. It is worth noting that the relationship between risk-taking impulsivity and its neurobiological markers may occur independently of dopamine, in contrast to the observed association in delay discounting impulsivity. For instance, bupropion, an antidepressant that inhibits reuptake of norepinephrine and dopamine, was found to enhance attention but does not appear to influence performance on the BART (Acheson & de Wit, 2008). Our recent findings suggest an association between risk-taking impulsivity and suicide that is modulated by the temporal dynamics of suicidal events through the upregulation of sensory brain regions, especially the postcentral gyrus, precuneus, and insula (Lee et al., 2024). Although these findings appear promising, the BART is not an optimal measure of general impulsivity, but rather of risk-taking propensity in reward-seeking. Moreover, while the BART shows reasonable test–retest reliability (Buelow & Barnhart, 2018; White et al., 2008), it demonstrates moderate to poor reliability in eliciting brain activity (Korucuoglu et al., 2020; Li et al., 2020).

In sum, studies exploring the link between risk-taking impulsivity and suicide risk have obtained mixed results, mainly due to the lack of psychometric cohesion between measures of risk-taking impulsivity. While risk-taking impulsivity may play a role in driving suicidal behavior, possibly by suppressing the brain’s regulatory functions (particularly those originating in the dorsolateral prefrontal cortex [dlPFC]), the relationship between risk-taking impulsivity and suicide risk is complex and may vary across different suicidal contexts. Further research is needed to better understand these relationships and their implications for suicide research.

##### Decision impulsivity: Reflection impulsivity

Reflection impulsivity is the tendency to make decisions prematurely, often without sufficient evidence (Kagan, 1966). Some studies have shown that tasks measuring reflection impulsivity, such as the Beads Task and Iowa Gambling Task, might inform our understanding of suicide risk. In the Beads Task, a degree of reflection impulsivity is inversely measured by how much participants seek evidence prior to decision-making. Simulation results, for example, suggest that reflection impulsivity is related to the average reward gained from the task, and this relationship is modulated by environmental uncertainty (Burk & Averbeck, 2023). However, the only study to use the Beads Task to investigate the relationship between reflection impulsivity and history of suicide attempts found no association (Bjork et al., 2022).

Mixed results have been obtained when using the Iowa Gambling Task to investigate the relationship between reflection impulsivity and suicide risk. In the Iowa Gambling Task, participants select advantageous or disadvantageous decks over repeated trials. Individuals with a history of suicide attempts have been reported to exhibit both better (Pan, Segreti, et al., 2013b) and worse (Bridge et al., 2012; Dombrovski et al., 2019) performance compared with clinical controls without such a history or healthy volunteers. Better performance on the Iowa Gambling Task seems driven by an increased tendency for risk-seeking strategies (Pan, Segreti et al., 2013b). Conversely, worse performance on the task is potentially related to an individual’s learning ability (Bridge et al., 2012; Dombrovski et al., 2019), possibly due to decreased value-based expectation to reward-oriented decision-making, driven by the ventromedial prefrontal cortex (vmPFC; Dombrovski et al., 2013). These findings suggest that the relationship between performance on the Iowa Gambling Task and suicide may involve two distinct mechanisms: hypersensitivity to and reduced expectation or learning capacity from incentives or emotional cues. These mechanisms could contribute to maladaptation to incentive or emotional cues, ultimately increasing the likelihood of making premature decisions, driven by brain regions associated with emotion regulation (dlPFC and vmPFC) and reward processing (striatum; Brown et al., 2020; Martin & Delgado, 2011; Zhao et al., 2021). For example, one study recruited female volunteers and asked them to play the Iowa Gambling Task immediately following passive viewing of emotional faces (Alacreu-Crespo et al., 2020). They specified the concordance condition as choosing the safe deck following positive emotional face cues and the risky deck following negative emotional face cues. The discordance condition was the opposite. The results showed that, in the concordance trials, individuals with a history of suicide attempts had reduced brain activation in the ventrolateral PFC (vlPFC) that was only observed in the early phase of the task. Such results suggest that a history of suicide attempts may be inversely related to the expected emotion regulation driven by reduced activation of the vlPFC. This, in turn, suggests that a history of suicide attempts may be associated with altered emotion regulation processes during decision-making tasks involving emotional cues.

There may be no direct relationship between reflection impulsivity and suicide risk at the behavioral level (Banca et al., 2016; Bjork et al., 2022; Gorlyn et al., 2013; Ho et al., 2018; Wyart et al., 2016). For instance, a recent meta-analysis found that suicide attempters tended to make riskier decisions on the Iowa Gambling Task or Cambridge Gambling Task, though the effect size remained modest, and that the lethality of the means in their previous attempts appeared to influence performance (Perrain et al., 2021). These findings emphasize the need for alternative measures, such as neuroimaging markers, to better understand this association. In addition, while previous research has focused on using reward-related behaviors to estimate reflection impulsivity, there is a need to use biologically salient information, such as emotional and sensory stimuli, to provide a more comprehensive understanding of the information on this topic.

##### Reward-based learning in suicide

Moment-to-moment reward-based learning has also been linked to suicide risk, though it remains a relatively underexplored area in research. Previous reviews have proposed that suicide can be better understood through the lens of pain, stress, and reward-related learning mechanisms, particularly those involving incentive sensitization and negative reinforcement learning (Elman et al., 2013). These mechanisms can lead to reward-based decision-making processes (Martin & Delgado, 2011).

Dombrovski and Hallquist (2022) reviewed the reinforcement learning model within the context of suicide and established a framework for understanding how reward-based learning, given multiple options, relates to the processes leading to suicide (Dombrovski & Hallquist, 2022). They suggested that failures in reward-based learning, due to limited cognitive capacity and dysregulated cognitive control, could increase suicide risk. In the three-armed bandit task, individuals with depression and a history of suicide attempts took longer to make decisions and demonstrated lower accuracy in distinguishing between the best and alternative options compared with those without such a history (Dombrovski et al., 2019). These individuals may have difficulty learning from experience and making optimal choices based on their estimations, suggesting that impaired reward-based learning may be associated with depression and suicide risk.

Interestingly, in the probabilistic learning task, learning rates for the gain condition, estimated using the reinforcement learning model, were higher in the context of negative emotion induction than positive emotion induction in individuals with BPD (Dixon-Gordon et al., 2022). In that study, suicide risk was positively related to learning rate during the test phase, especially in the negative emotion induction condition. While learning accuracy for the gain condition was higher in the positive emotional induction condition, suicide risk did not mediate this relationship. This suggests the potential utility of the reinforcement learning model in the context of suicide and emotion processing. This model extends beyond reflection impulsivity to include inflexible decision-making, with a specific focus on brain regions involved in sensory and emotion regulation, including the vlPFC and dlPFC (Schmaal et al., 2020). Interestingly, a recent paper proposed a computational framework investigating inferential cognitive processing that incorporated learning rates and other potential markers, such as belief decay rate (as a measure of memory) and stress-oriented markers (measured by stress reactivity and controllability) in simulating go/no-go task performance (Karvelis & Diaconescu, 2022). The framework suggests that these processes lead to behavioral changes such as hopelessness and cognitive biases; it also suggests that norepinephrine-driven connections between the locus coeruleus, amygdala, dorsal PFC, and ACC, along with serotonin-driven networks between the dorsal raphe nucleus and vmPFC, may be responsible for inferential cognitive processing. Further research is required to explore the association between learning parameters, emotional and sensory stimuli as inputs, and suicide risk, taking into consideration the brain regions responsible for sensory and emotion regulation.

The parameters surrounding reward-based learning offer valuable insights into understanding mental disorders. In particular, it appears that learning parameters estimated from reinforcement learning in task-driven, reward-based learning can function as markers for individual learning capability and decision tendencies, potentially acting as risk factors for mental disorders (Huys et al., 2021; Zhang et al., 2020). In the context of depression, rates of learning have been negatively associated with anhedonia and negative affect in reward learning and reward loss conditions, respectively (Brown et al., 2021). Compared with healthy volunteers, individuals with depression also exhibited reduced precision in decision-making when lower reward probabilities were assumed, and they exhibited higher learning rates for negative cues in the two-armed bandit task (Vandendriessche et al., 2023). Similarly, individuals with MDD or bipolar disorder both showed lower learning rates in a working memory task compared with healthy volunteers (Cheng et al., 2024), underscoring the relevance of reinforcement learning-derived parameters in understanding depression.

Establishing a direct link between suicide and reward-based learning estimated by reinforcement learning parameters remains a complex challenge. However, these studies provide valuable insights into the potential utility of tasks assessing value-based learning hyperparameters in mental health research, especially in suicide research, and their relationship to sensory and emotion regulation in the brain.

### Potential factors compounding the relationship between aggression, impulsivity, and suicide risk

It remains unclear how aggression and impulsivity, as single risk factors, may directly contribute to suicide risk. As noted above, both reactive aggression and various types of impulsivity have been associated with suicide risk. However, the HiTOP taxonomic categorization (https://www.hitop-system.org/current-model) classifies suicidality under the distress subset of the internalizing spectrum, partially related to mood and anxiety disorders. In contrast, aggression and impulsivity are categorized under the disinhibited externalizing subset of the externalizing spectrum, partially linked to antisocial personality disorders (Conway et al., 2022; Kotov et al., 2018). This framework suggests that other distress components of the internalizing spectrum may compound the relationship between reactive aggression and suicide risk.

Negative affect, specifically irritability, may also compound the relationship between aggression, impulsivity, and suicide. Serotonergic system-driven impulsivity may act as a diathesis, establishing a foundation whereby aggression may contribute to suicide risk (Siever, 2008). Previous studies found that reactive aggression and negative affect are positively correlated (Achterberg et al., 2016; Shields et al., 2024), and other studies suggest that negative affect can drive suicidal thoughts, behaviors, and nonsuicidal self-injury (Allen et al., 2022; Erbuto et al., 2018; Nicolai et al., 2016; Orme et al., 2020; Rigucci et al., 2021; Rubio et al., 2020), especially in individuals with BPD (Mou et al., 2018). One specific state of negative affect, irritability, has been linked to reactive aggression (Evans & Burke, 2024), manifesting as dysfunction in frontal brain regions (DeYoung & Allen, 2019). Finally, other studies found that irritability contributes to suicide risk (Jha et al., 2020, 2021; Liu et al., 2021), suggesting that the relationship between irritability and suicide risk warrants further exploration.

Hyperarousal, another distress component of the internalizing spectrum, may also compound the relationship between aggression, impulsivity, and suicide risk. Hyperarousal has been positively associated with impulsivity (Eysenck, 1987; Zhang et al., 2015) and suicide risk (Morabito et al., 2020) and may also interact with previous adverse life events in the manifestation of suicidal behaviors (Kwon et al., 2021; Stanley et al., 2019b), with the relationship being particularly pronounced in individuals with mood disorders (Dolsen et al., 2017). Hyperarousal may drive individuals with suicide risk to escape from environmental stressors (Chiurliza et al., 2018) while modulating the autonomic nervous system (Calati et al., 2020), ultimately leading to suicidal behaviors. Future studies should seek to determine whether irritability and hyperarousal modulate the relationship or are independently linked to suicide risk.

Age and biological sex are also important factors to consider in the relationship between aggression, impulsivity, and suicide risk. For instance, the positive association between aggression and impulsivity and suicide risk appears to be negatively moderated by age (Conner et al., 2004; McGirr et al., 2008). The relationship between suicide risk and behavioral traits, including impulsivity and physical forms of aggression, appears to be more pronounced in males (Horesh et al., 1999; McGlade et al., 2021; Menon et al., 2015). Additional research is needed to clarify the mechanism though which age and biological sex influence the relationship between aggression, impulsivity, and suicide risk.

### Heterogeneity of suicide risk factors

The heterogeneity of suicide risk factors contributes to the association between aggression, impulsivity, and suicide risk; in this context, the lethality of suicide attempts and cognitive rigidity are critical factors in understanding this relationship. Several studies noted associations between aggression, impulsivity, and both the past and future lethality of suicide attempts (Baca-García et al., 2001; Gvion, 2018; Gvion et al., 2014). Recent research indicates that aggression is linked with high-lethality suicide attempts, while impulsivity tends to be associated with low-lethality suicide attempts (Brokke et al., 2022), effects that may be potentially negatively mediated by age (Doherty et al., 2023). Cognitive rigidity has also been associated with impulsivity and suicidal thoughts (Morris & Manseli, 2018; Novak et al., 2022; Rutter et al., 2020). Interestingly, the lethality of suicide attempts and cognitive rigidity may collectively help subtype individuals. For example, individuals with high-lethality suicide attempts tend to exhibit inconsistent valuation, along with deficits in learning and cognitive control, rather than a simple preference for delay discounting (Tsypes et al., 2022). This indicates that a rigid and narrow style of value-based decision-making may be more characteristic of those with high-lethality profiles. In contrast, individuals with low-lethality suicide attempts often display excessive shifts in preferences across trials, regardless of whether the preference was rewarded (Tsypes et al., 2024). These findings suggest that different patterns of value-based decision-making—rigid versus excessive—may manifest depending on the lethality of the suicide attempt. Echoing the aggression and impulsivity subtypes reviewed earlier, the rigid and excessive subtypes of value-based learning could potentially disrupt emotion regulation (Pruessner et al., 2020), which is closely tied to the relationship between aggression, impulsivity, and suicide risk. Additional research is needed to validate the mechanisms underlying these association.

Interestingly, Rimkeviciene and De Leo (2015) categorized impulsive suicide risk factors into four subtypes:

1. Time-related criteria;
2. Absence of proximal planning for suicidal events;
3. Presence of proximal/distal planning of suicidal events, and
4. Other factors.

This framework can be simplified into

1. The absence of planning for suicidal events and
2. The temporal dynamics of suicidal events.

A handful of studies have explored the association between aggression, impulsivity, and planned or unplanned suicidal events. For instance, in criminal populations, social aggression appears to be associated with planned, but not unplanned, suicide attempts (Swogger et al., 2014b). Individuals with planned suicide attempts also tend to exhibit severe and frequent suicidal thoughts, a family history of suicide completion, and increased motor impulsivity (Chaudhury et al., 2016). However, additional research is needed to clarify how planning suicidal behaviors may or not may influence the relationship between aggression, impulsivity, and suicide risk.

In addition, investigating the relationship between aggression, impulsivity, and suicide requires careful consideration of the temporal dynamics of suicidal events. Previous research suggests that lifetime history of suicide attempts may be too distal to be associated with ongoing neurobiological markers, such as somatosensory measures of the brain (Rabasco & Andover, 2020). Thus, it is crucial to consider the temporal dimension when measuring individual-level suicidal behavior (Abed & St John-Smith, 2022; Corke et al., 2021), both from a cognitive and neurobiological perspective. One study found that cognitive performance, including response inhibition and memory recognition, was linked to the recency of past suicide attempts (Interian et al., 2020). Specifically, individuals who had attempted suicide within the past week displayed greater cognitive impairments than those with no suicide attempts within the past year. Another study observed a positive correlation between lifetime suicide attempts, lifetime suicidal thoughts, and dissociation, but no association was found between lifetime suicide attempts and current pain tolerance, suggesting that past suicidal behaviors may not accurately reflect present somatosensory measures (Rabasco & Andover, 2020). Another study of young adults experiencing recent suicidal thoughts found that they were more likely to disengage from suicide-specific cues faster than individuals with a lifetime history of suicidal thoughts (Rosario-Williams et al., 2023). These studies underscore the importance of considering the recency of suicidal behaviors when investigating cognitive and neurobiological variables.

The relationship between aggression, impulsivity, and suicide risk is influenced by temporal dynamics. Ecological momentary assessment (EMA) has been shown to capture moment-to-moment fluctuations in suicide risk (Ballard et al., 2021), suggesting that impulsivity, as a marker of suicide risk, may vary over time in relation to suicidal thoughts. Some studies indicate that impulsivity involves a multi-factor structure, which can be treated as a trait-like construct when averaged and correlated with factors such as capacity/capability for suicide (Hadzic et al., 2020; Halvorson et al., 2021). While most of the variability in impulsivity arises from between-individual trait differences, a small amount of variability stems from moment-to-moment fluctuations (Halvorson et al., 2021). These fluctuations in impulsivity are associated with changes in suicidal thoughts over time (Luise et al., 2022) and with a history of suicide attempts, particularly during periods of suicidal crisis (Millner, Lee et al., 2020a). However, one study found that impulsivity did not seem to vary across time in individuals with bipolar disorder (Santana et al., 2022); this lack of variability might reflect that the study assessed impulsivity within narrow timeframes, such as during manic episodes when impulsivity is heightened. Extending the measurement period to include inter-episodic states, such as manic and depressive phases, could reveal temporal dynamics in impulsivity that are currently overlooked, and a broader temporal lens might reveal variations in impulsivity that align with the episodic nature of bipolar disorder.

As noted above, risk-taking impulsivity is linked to suicidal behavior (Dougherty et al., 2009) but, notably, its association may vary based on the temporal dynamics of the suicidal crisis (Cole et al., 2019). For example, when using impulsivity as a factor to predict suicidal behavior, no difference was observed between individuals with current suicidal thoughts and those with recent suicide attempts within the past 2 weeks (Millner, Robinaugh et al., 2020b). In addition, two previous studies examining cognitive performance on the Iowa Gambling Task among adolescents who had previously attempted suicide yielded mixed results. The discrepancy could be attributed to differences in participant recruitment: one study focused on individuals who had experienced a suicidal crisis within the past year (Bridge et al., 2012), while the other included participants with a lifetime history of suicide attempts (Pan, Hassel et al., 2013a). Collectively, these results underscore the importance of considering the recency of suicidal behavior when investigating the link between impulsivity and suicidal behaviors.

In summary, the heterogeneity of suicide risk factors surrounding the planning and temporal dynamics of suicidal events may complicate the association between aggression, impulsivity, and suicide risk. Although the current evidence is limited, planning of suicidal events could reflect different profiles of aggression and impulsivity. In addition, the temporal dimension of aggression has been relatively understudied. Though previous research suggests a potential link between the timing of aggressive and suicidal behaviors (Deffenbacher et al., 1996; van Gelder et al., 2022), the exact relationship remains uncertain.

### Genetic and immune markers and transmitters for aggression and impulsivity

The stress system, in combination with aggression and impulsivity, has been closely linked with suicide risk. Cortisol levels, a quasi-index of stress system activity governed by the HPA axis, are associated with aggressive behaviors (Böhnke et al., 2010). One review found that elevated cortisol levels were related to reactive aggression, whereas reduced cortisol levels were linked to proactive aggression (Barzman et al., 2010). Similarly, another study found that suicide attempters with high levels of reactive aggression had increased cortisol reactivity during the TSST (Stanley et al., 2019a). However, the direction of the association between HPA axis activity, aggression, and impulsivity is complicated, particularly when considering developmental factors (Gollan et al., 2005; Walker et al., 2018), suggesting a nonlinear relationship.

Moreover, dysregulation of the HPA axis, viewed as chronically elevated cortisol levels or inadequate suppression in dexamethasone suppression tests, has been directly tied to suicide risk (Berardelli et al., 2020; Jokinen & Nordström, 2009; Jokinen et al., 2010). This suggests that the stress system, potentially interacting with noradrenergic activity, is intricately connected to suicide risk (Jokinen et al., 2010; Melhem et al., 2016). Genetic studies add another layer, especially for corticotropin-releasing hormone receptor 1 (*CRHR1*). For instance, *CRHR1* variants have been shown to interact with life stressors to predict suicide attempts (Breen et al., 2015; Ludwig et al., 2018; McGirr et al., 2010; Roy et al., 2012), and a Swedish study found that *CRHR1* variants, combined with a history of childhood physical abuse, increased the risk of suicide attempts, particularly in individuals with a family history of suicide attempts (Ben-Efraim et al., 2011). These findings underscore the multifaceted role of the stress system, primarily measured through HPA axis activity, in mediating the relationship between aggression, impulsivity, and suicide risk.

The immune system has also been linked to aggression and impulsivity. Aggression has been associated with increased inflammatory cytokine levels and dysregulated immune response (Takahashi et al., 2018). These immune changes are thought to be modulated by microglial activity (Takahashi, 2024), highlighting a potential neuroimmune mechanism underlying aggression and, potentially, impulsivity. Although limited research exists regarding the immune system’s role in aggression, impulsivity, and suicide, a few studies have considered the association between *Toxoplasma gondii* (T. gondii) infection and these risk factors (Bak et al., 2018; Cook et al., 2015; Hamid et al., 2022; Soleymani et al., 2020). However, recent studies failed to find such an association in prison inmates (Rocha-Salais et al., 2023) or decedents (Mendoza-Larios et al., 2023), suggesting that further research is needed to validate the association. In a preclinical study using a mouse model, social stress was found to modify immune-related gene expression, such as histone deacetylase 1 (*HDAC1*), methyl CpG binding protein 2 (*MeCP2*), and ten-eleven translocation (*TET*) mRNA expression in mice exhibiting high levels of aggression (Matrisciano & Pinna, 2021). These gene modifications are thought to contribute to reduced energy metabolism and increased inflammation in the hippocampus, potentially linking aggression with stress-induced metabolic and immune dysregulation. The findings suggest that aggression and impulsivity may interact with the immune system to influence suicide risk and its neural correlates, but more research is needed to validate the mechanistic association between aggression, impulsivity, and suicide risk through immune-related processes.

Neurotransmitters, including serotonin, glutamate, GABA, norepinephrine, dopamine, neuroinflammation, neurotrophins, cholesterol, fatty acids, and hormones have also all been studied in relation to aggression, impulsivity, and suicide risk. While a comprehensive review of these neurotransmitters is available elsewhere (Mann & Rizk, 2020; Van Heeringen, 2018), the findings regarding serotonin, glutamate, and neurosteroids (especially testosterone)—and their relevance to suicidal behavior—is briefly summarized below.

Extensive research has explored the relationship between serotonin and its role in aggression, impulsivity, and suicide risk. Reduced serotonergic function has been linked to increased aggression and impulsivity, potentially via regulation of dominance in social hierarchies (Carver et al., 2008; Dolan et al., 2001). Such serotonergic dysfunction may function as a diathesis that contributes to the interaction between aggression, impulsivity, and suicide risk (Underwood & Mann, 2003). For instance, initial postmortem studies revealed lower levels of serotonin in the spinal stem and lower levels of its metabolite (5-HIAA) in the hindbrain of individuals who died by suicide (Bourne et al., 1968; Pare et al., 1969). A small, early study found a positive relationship between lifetime aggressive behaviors and a history of suicide attempts alongside a negative correlation with 5-HIAA levels in cerebrospinal fluid (CSF; G. L. Brown et al., 1982). Similarly, a study of outpatients with personality disorder found an inverse correlation between lifetime aggression and platelet serotonin levels (Goveas et al., 2004). Serotonergic dysfunction in brain regions implicated in executive control, such as the PFC and ACC, have also been hypothesized to contribute to the onset of aggressive behavior (Seo et al., 2008). Another study found that female youths undergoing acute tryptophan depletion, which temporarily lowers serotonin levels, exhibited increased impulsive behaviors and increased attentional capacity during the Matching Familiar Figures Test (Fikke et al., 2013). These findings suggest that reduced serotonin levels may potentially contribute to an elevated risk of suicidal behaviors. Conversely, another study of individuals with personality disorders found high levels of aggression and impulsivity but no association with CSF 5-HIAA levels (Simeon et al., 1992). Menon and Kattimani speculated that these mixed results could indicate upregulation of 5-HT1A autoreceptors in the dorsal raphe nucleus (Manon & Kattimani, 2015). Such upregulation of autoreceptors would decrease serotonin release and activity, potentially triggering a homeostatic upregulation of bodily functions to enhance serotonin bioavailability. However, evidence for this hypothesis is currently lacking.

Glutamatergic and GABAergic biomarkers have also emerged as potential risk factors in the interplay between aggression, impulsivity, and suicide risk. Previous studies found that GABA levels in the ACC were inversely correlated with aggression and impulsivity in individuals with attention-deficit/hyperactivity disorder (ADHD; Ende et al., 2016). Another study found that CSF glutamate levels were positively associated with reactive aggression (Coccaro et al., 2013). These studies suggest that the balance between glutamate and GABA may be key to regulating aggression and impulsivity, particularly within the ACC (Chaibi et al., 2023; Siever, 2008). Although direct evidence linking the glutamatergic and GABAergic systems to the interaction between aggression, impulsivity, and suicide risk is lacking, previous research identified relationships between these systems and suicide risk (for detailed reviews, see Fiori & Turecki, 2012; Turecki et al., 2019). Briefly, postmortem studies reported changes in glutamatergic and GABAergic gene expression in suicide victims, mostly in prefrontal regions of the brain, though further exploration into their ability to mediate aggression and impulsivity in individuals at risk of suicide is needed. A spectroscopy study also found higher levels of glutamate and glutamine metabolites in the ACC of individuals with depression and suicidal thoughts compared with those with depression only (Lewis et al., 2020). These findings suggest that dysregulation of glutamatergic transmission and synthesis in prefrontal regions may a key risk factor in suicide. Interestingly, growing research into the N-methyl-D-aspartate (NMDA) receptor antagonist ketamine—known for its acute therapeutic effects in depression and suicidal thoughts (Murrough et al., 2015; Singh et al., 2024; Wilkinson et al., 2018; Zarate et al., 2006)—has provided additional insights into the relationship between glutamate and suicide. A recent study found that the glutamatergic modulator ketamine both induced antidepressant effects and reduced suicidal thoughts, suggesting that it may reduce suicide risk either independently (Ballard et al., 2014) or by modulating mood mechanisms (Hochschild et al., 2022). These mechanisms could be even more comprehensive when considering their impact on aggression and impulsivity; for instance, a recent review suggested that ketamine’s antisuicidal effects may be related to its ability to modulate aggression and impulsivity via serotonergic, neuroinflammatory, and stress systems (Nguyen et al., 2023). Further investigation is needed to clarify these effects.

Finally, there is a potential relationship between suicidal behavior and neurosteroids, especially testosterone, a sex hormone that induces anabolic and androgenic bodily changes in men (Tyagi et al., 2017) and also functions as a social hormone (Rice & Sher, 2015). Previous studies found that exogenous testosterone administration can induce aggressive behaviors in men with high social rank and high impulsivity and impulsive decision making (Carré et al., 2017; Wu et al., 2020, 2022). Chronic testosterone administration has been linked to increased impulsivity, potentially mediated through the adrenergic receptor-protein kinase A (PKA) signaling pathway in the PFC (Agrawal et al., 2019). Interestingly, testosterone administration appears to affect different facets of impulsivity. For instance, testosterone administration can enhance risk tolerance without impacting motor impulsivity (Cooper et al., 2014). Moreover, a recent study found that the interaction between testosterone and impulsivity is potentially mediated by other systems—including the GABAergic system—warranting a multisystemic approach to understanding the relationship between aggression, impulsivity, and testosterone. It is important to note that the behavioral effects of testosterone are highly context dependent (Carré & Archer, 2018), suggesting that the relationship between testosterone, aggression, and impulsivity should be explored within the specific social and environmental context of the individual. Testosterone has also been proposed as a mediator of the effects of impulsivity and aggression on suicidality (Sher, 2017). As an example, suicide attempts were found to be positively related to testosterone levels in males but not females (Stefansson et al., 2016); the same study found that, in males with a history of suicide attempts, the testosterone/cortisol ratio score was positively related to impulsivity and aggression. Another study found that baseline testosterone levels predicted subsequent suicide attempts in males with bipolar disorder (Sher et al., 2022). Further investigation is warranted to validate the relationship between testosterone and suicide risk markers, aggression, and impulsivity.

The complex interplay between biological stress and immune markers, as well as neurotransmitters, can contribute to suicide risk. This interplay needs further investigation, particularly in the context of aggression and impulsivity. Understanding these relationships is critical for identifying potential suicide biomarkers and developing prevention and intervention strategies in suicide research.

## Summary and future directions

This review explored the relationship between aggression, impulsivity, and suicide. While aggression and impulsivity have been associated with suicide risk, any mechanistic understanding of these associations requires further investigation. Reactive aggression, which may be influenced by sensory and emotion processes in specific brain regions, could be associated with suicide risk, particularly when considering biological sex and emotional arousal. Self-reported impulsivity, related to self-reflection and emotion regulation, also requires further validation. Response inhibition is also associated with suicide risk resulting from neural dysfunction in brain regions related to motor response and its regulation. The relationship between suicide risk and different types of task-driven decision impulsivity—including delay discounting impulsivity (governed by sensory and regulatory function in the parahippocampal gyrus, occipital gyrus, and lateral PFC) and risk-taking impulsivity (potentially driven by inhibitory functions in the frontal cortex and insula)—are inconsistent and multifaced. Currently, the relationship between suicide risk and reflection impulsivity (associated with decision-making regions in the frontal and parietal areas) remains understudied.

Although previous research identified potential neural markers of aggression and impulsivity, more studies are needed to understand how aggression and impulsivity contribute to cumulative suicide risk at a cognitive level. Future research should explore how impulsivity and aggression impact cognitive capacity, cognitive control, and reward-based learning processes, which may manifest as suicidal behaviors. Incorporating currently understudied measures, such as reflection impulsivity and learning parameters estimated by the reinforcement learning model, is essential. While establishing a direct link between suicide, reflection impulsivity, and decision-making parameters presents a complex challenge, the studies reviewed above offer valuable insights into the potential utility of these measures in suicide research, promising to enhance our understanding of suicide risk and its related behaviors.

Moreover, the heterogeneity of suicide risk factors, particularly the planning and temporal dynamics surrounding suicidal events must also be taken into account. For example, planned suicidal behaviors are linked to specific traits such as social aggression and increased motor impulsivity, while unplanned suicidal behaviors may reflect distinct psychological processes. Understanding the temporal dynamics of suicidal events and their interaction with aggression and impulsivity is crucial for disentangling the complex interconnections between aggression, impulsivity, and suicide risk. The recency of suicidal behavior may influence how aggression and impulsivity contribute to suicide risk, as recent suicidal attempts or thoughts could amplify or modulate these traits. Neuroimaging markers and biomarkers, real-time monitoring methods like EMA, and longitudinal study designs can capture the temporal dynamics of suicidal behavior at multiple levels (Halvorson et al., 2021; Luise et al., 2022). Stress system-related genetic predisposition, such as CRH gene receptor polymorphisms, proinflammatory markers, and dysregulation of neurotransmitters, including serotonin, glutamate, GABA, and testosterone, may contribute to suicide risk, as well as to the relationship between suicide risk, aggression, and impulsivity. Further research is needed to unravel these associations.

Finally, it should be noted that the suicide risk biomarkers discussed in this review are inherently multifaceted and operate at multiple levels. While this review synthesizes findings related to aggression, impulsivity, and suicide risk, the findings are often inconsistent or contradictory even within the same subtype of aggression or impulsivity. This lack of coherence may stem not only from experimental biases or differences between samples but also from the absence of psychometric cohesion in the tools used to assess these constructs. Furthermore, reducing variability in how aggression and impulsivity are categorized could potentially yield more robust and insightful interpretations of the results (Sharma et al., 2014). A cautious interpretation of current studies is essential, and further refinement in both conceptual frameworks and measurement techniques is needed to draw more definitive conclusions.

Moving forward, three pressing areas of research need to be addressed in the quest to unravel the complexities surrounding suicide. First, it is important to explore the extent to which aggression and impulsivity affect sensory and emotion regulation as well as the decision-making process. Second, effective methods are needed to accurately capture the processes involved in sensory and emotion regulation in decision-making processing, measured by reflection impulsivity and learning parameters. Third, such research should occur via longitudinal studies, which offer a rigorous scientific framework for examining suicide-related factors over an extended period and allow findings to be both validated and replicated. These key areas of future research have the potential to provide a more comprehensive understanding of the intricate connections that underlie suicidal behaviors and their broader implications in the context of mental health and suicide prevention.

## Conclusion

This review has highlighted the importance of biological factors related to impulsivity and aggression and their link to suicide risk. Emotional sensitivity, sensory and emotion regulation, and their neural correlates are key to understanding suicide risk. The relationship between suicide and various subtypes of aggression and impulsivity is multifaced, compounded by factors such as age, biological sex, distinct profiles of decision-making processes, and potentially alternative factors, such as irritability and hyperarousal. These complexities emphasize the importance of precisely defining and measuring these constructs. Moreover, incorporating considerations of prior planning and the temporal dynamics of suicidal events into the assessment of diatheses, stressors, and biopsychological risk factors is vital for advancing suicide research. Reflection impulsivity, reinforcement learning parameters, and emotional and sensory regulation are potentially useful areas for future study. Additional research is needed to expand our understanding of the intricate connections underlying suicidal behaviors, aggression, and impulsivity, with the ultimate goal of improved identification and treatment of at-risk individuals.

## Data Availability

Not applicable.
